# What can we learn about the psychiatric diagnostic categories by analysing patients' lived experiences with Machine-Learning?

**DOI:** 10.1186/s12888-022-03984-2

**Published:** 2022-06-24

**Authors:** Chandril Chandan Ghosh, Duncan McVicar, Gavin Davidson, Ciaran Shannon, Cherie Armour

**Affiliations:** 1 School of Psychology, Queen’s University Belfast, Belfast, United Kingdom; 2 Queen’s Management School, Queen’s University Belfast, Belfast, United Kingdom; 3 School of Social Sciences, Education and Social Work, Queen’s University Belfast, Belfast, United Kingdom; 4 IMPACT Research Centre, Northern Health and Social Care Trust, Antrim, United Kingdom

**Keywords:** Classification, Taxonomy, Machine Learning, Lived experiences, Narratives

## Abstract

**Background:**

To deliver appropriate mental healthcare interventions and support, it is imperative to be able to distinguish one person from the other. The current classification of mental illness (e.g., DSM) is unable to do that well, indicating the problem of diagnostic heterogeneity between disorders (i.e., the disorder categories have many common symptoms). As a result, the same person might be diagnosed with two different disorders by two independent clinicians. We argue that this problem might have resulted because these disorders were created by a group of humans (APA taskforce members) who relied on more intuition and consensus than data. Literature suggests that human-led decisions are prone to biases, group-thinking, and other factors (such as financial conflict of interest) that can enormously influence creating diagnostic and treatment guidelines**.** Therefore, in this study, we inquire that if we prevent such human intervention (and thereby their associated biases) and use Artificial Intelligence (A.I.) to form those disorder structures from the data (patient-reported symptoms) directly, then can we come up with homogenous clusters or categories (representing disorders/syndromes: a group of co-occurring symptoms) that are adequately distinguishable from each other for them to be clinically useful. Additionally, we inquired how these A.I.-created categories differ (or are similar) from human-created categories. Finally, to the best of our knowledge, this is the first study, that demonstrated how to use narrative qualitative data from patients with psychopathology and group their experiences using an A.I. Therefore, the current study also attempts to serve as a proof-of-concept.

**Method:**

We used secondary data scraped from online communities and consisting of 10,933 patients’ narratives about their lived experiences. These patients were diagnosed with one or more DSM diagnoses for mental illness. Using Natural Language Processing techniques, we converted the text data into a numeric form. We then used an Unsupervised Machine Learning algorithm called K-Means Clustering to group/cluster the symptoms.

**Results:**

Using the data mining approach, the A.I. found four categories/clusters formed from the data. We presented ten symptoms or experiences under each cluster to demonstrate the practicality of application and understanding. We also identified the transdiagnostic factors and symptoms that were unique to each of these four clusters. We explored the extent of similarities between these clusters and studied the difference in data density in them. Finally, we reported the silhouette score of + 0.046, indicating that the clusters are poorly distinguishable from each other (i.e., they have high overlapping symptoms).

**Discussion:**

We infer that whether humans attempt to categorise mental illnesses or an A.I., the result is that the categories of mental disorders will not be unique enough to be able to distinguish one service seeker from another. Therefore, the categorical approach of diagnosing mental disorders can be argued to fall short of its purpose. We need to search for a classification system beyond the categorical approaches even if there are secondary merits (such as ease of communication and black-and-white (binary) decision making). However, using our A.I. based data mining approach had several meritorious findings. For example, we found that some symptoms are more exclusive or unique to one cluster. In contrast, others are shared by most other clusters (i.e., identification of transdiagnostic experiences). Such differences are interesting objects of inquiry for future studies. For example, in clear contrast to the traditional diagnostic systems, while some experiences, such as auditory hallucinations, are present in all four clusters, others, such as trouble with eating, are exclusive to one cluster (representing a syndrome: a group of co-occurring symptoms). We argue that trans-diagnostic conditions (e.g., auditory hallucinations) might be prime targets for symptom-level interventions. For syndrome-level grouping and intervention, however, we argue that exclusive symptoms are the main targets.

**Conclusion:**

Categorical approach to mental disorders is not a way forward because the categories are not unique enough and have several shared symptoms. We argue that the same symptoms can be present in more than one syndrome, although dimensionally different. However, we need additional studies to test this hypothesis. Future directions and implications were discussed.

**Supplementary Information:**

The online version contains supplementary material available at 10.1186/s12888-022-03984-2.

## Introduction

Diagnostic categories are important for mental healthcare services and research. Current diagnostic approaches have been demonstrated to be unreliable [1–3], and their usefulness questionable [4] because it is unable to clearly differentiate between different service seekers (i.e., between-disorder diagnostic heterogeneity). So, developing an alternative diagnostic approach is warranted and arguably necessary to advance research and clinical practice further. In this study, we propose an alternative way to categorise psychopathological symptoms.

The end goal of healthcare is to minimise or remove harmful or unhealthy experiences and promote well-being. The grouping of symptoms or diagnostic categories is important to any branch of health care, including mental health. It facilitates clear and consistent communication with patients, physicians, the government, and other stakeholders. It also facilitates the development of a treatment that would otherwise be difficult to develop as well as to administer. Concerning mental ill health, psychiatry has created diagnostic categories for patients’ experiences. However, the existing approaches have been previously criticised for a range of limitations, including being “shrouded in the rhetoric of science” [5].

### Problems with the traditional taxonomies, the DSM and the ICD

There is an emerging literature suggesting that the traditional diagnostic systems such as the Diagnostic and Statistical Manual of Mental Disorders (DSM–5, [6]) and the International Classification of Diseases 11th Revision (ICD-11, World Health Organization, WHO, 2020) [7] are unreliable (e.g.,[1, 8, 9]). The same service seeker might receive two different diagnoses by two independent clinicians (i.e., low inter-rater reliability). In other words, two or more people (e.g., clinicians) do not arrive at the same diagnosis given an identical set of data. For example, one of the studies demonstrated that 40% of diagnoses did not meet even a relaxed cut-off for acceptable interrater reliability [9]. Two related concerns (as reviewed in [9] are that there is a co-occurrence of symptoms between disorders suggesting an excessive overlap of symptoms between people who received different diagnoses. The second concern is that some patients’ experiences do not fit neatly with the disorders’ criteria. This indicates that some people, despite expressing significant distress or impairment and need for help, do not fit well with the criteria for the DSM’s diagnostic categories.

The root of these problems may relate to the fact that historically such disorders were derived using a top-down approach where a committee of experts agreed upon certain names of disorders and which symptoms to be included for its diagnosis based on the ideologies prominent at that time of history [10]. As a result, these traditional diagnoses rely on certain untested assumptions, such as the assumption that mental disorders can be organised effectively into categories and which symptoms to include under what label.

Furthermore, there might have been undue political and commercial influences in creating the classification systems, like the Diagnostic and Statistical Manual of Mental Disorders (DSM-5), created by the American Psychiatric Association (APA) task force members. For example, it was reported that 69% of the members responsible for creating the DSM-5 had ties to the pharmaceutical industry [11]. It has been argued that such financial ties might have had an enormous influence on the diagnostic and treatment guidelines (e.g., “pro-industry habit of thought”). In other words, the DSM was not without conflict of interests.

Such unreliable diagnostics is problematic because such manuals shape the way healthcare professionals and the society at large views mental illnesses and those suffering from it. So, an unreliable diagnostic system is expected to lead to misjudge people experiencing psychopathology on how they are experiencing it, how much help they need, and what they are capable of – with important implications such as taking away their voting rights—as in the United Kingdom where patients can lose their right to vote “if deemed to lack mental capacity by a health care provider” [12]. Furthermore, unreliable diagnostics might lead to unreliable categorisation of research participants in clinical trials (e.g., anti-depressant trials) and other empirical studies (e.g., neuroimaging studies to differentiate brain functioning or studies attempting to find biomarkers in patients with a particular diagnosis from those people without that diagnosis) leading to production of questionable literature (knowledge base). Combined, an unreliable diagnostic system is likely to worse treatment. Likewise, due to the uncertainty in the validity of the current diagnostic categories, they do not always guide treatment and predict outcomes [13].

### Addressing the limitations of the current taxonomies

In the current study, the aims were to explore the possibility of an alternative diagnostic categorical system by taking a bottom-up approach where we built a diagnostic system from the narrative data of patients’ lived experiences. There was no limit on how many disorders or syndromes could be generated. There was also no initial decision about which symptoms would be included under each disorder. Instead, based solely on the structure of the data, the A.I. algorithm organised the data using 100 s of iterations performed using different symptoms. In simpler words, a single “iteration” refers to the step of estimating the centroid and assigning all the data points to the cluster based on their distance from the centroid. We have run several iterations to improve the quality of the clusters.

### Emerging alternative approach

One proposed an alternative way to understand psychopathology is the Hierarchical Taxonomy of Psychopathology (HiTOP]).

The HiTOP represents an emerging nosological system that organises psychopathology in a hierarchical format. The components in the higher levels of the structure indicate the most common or general features (shared between patients). They consist of dimensional syndromes as a continuum (or spectra). On the other hand, the components in the lower levels of the hierarchy (in the HiTOP model) consist of signs and symptoms specific to each condition/patient. The intermediate levels of HiTOP consist of subfactors, syndromes and components/traits in descending order. It is different from the DSM, as it does not categorise conditions. Instead, it attempts to allow for a flexible patient description depending on the desired degree of specificity.

Furthermore, the HiTOP has been able to inform the RDoC framework [14]. RdoC framework is a research framework for studying mental disorders. It aims to understand the nature of mental health and illness in terms of different degrees of dysfunction of the general psychological/biological system. The HiTOP has been argued to inform the RDoC framework [14] regarding key clinical dimensions that need to be considered and provide clearer phenotypes for basic research. HiTOP is a possible way forward in the post-DSM era, but it is yet to be adopted in mainstream practice.

### Problems with HiTOP

The HiTOP proposes a dimensional model, but clinical care often requires black-and-white(binary) decisions. The traditional taxonomies tend to offer a single cut-off, that is, the diagnostic threshold. The HiTOP, which follows a dimensional route, attempts to overcome this problem by segmenting dimensions into illness severity (like blood pressure ranges). This is similar to the clinical staging model framework that defines the extent of progression of a disorder at a time and where a person lies currently along the continuum of the course of a psychiatric condition such as Psychotic and Related Mood Disorders [15]. However, even when applied in practice, when compared with the DSM, HiTOP relatively complicates the communication process between different stakeholders of mental healthcare services.

Furthermore, it is important to note that both the HiTOP and the clinical staging model framework attempts to re-use many of the constructs from the traditional diagnostic systems. We argue that the focus should attempt on a symptom-level at this stage since the literature suggests that much of the constructs from the traditional diagnostic systems and literature (e.g., depression) suffers from low validity and low reliability. So, re-using such constructs would be repeating the same mistake.

We also argue that a limitation of those dimensions may be that they are based on self-report scales and questionnaires, which restricts the person’s responses to a set of conditions (set by the researcher) without allowing much room for reporting symptoms or experiences outside that pre-fixed list of symptoms and experiences. This risks loss of information (e.g., maybe the person had other more important symptoms to report, but it was not reported due to the restricted data collection tool). Therefore, a better alternative is to collect data from people using open-ended questions to report their phenomenological experiences.

Some people with relatively serious conditions might perceive and report their condition as less bothering. For example, some people might get habituated to the distress caused over the years and accept it as part of life. Others, such as patients who are also a parent, might be concerned that reporting their symptom severity honestly might lead to the professional judgement that there is an ongoing risk to the child from the parent with the possible outcome of children being removed into alternative care.

Simultaneously, some people might, consciously or unconsciously, exaggerate relatively minor concerns and report them as more disabling or severe. For example, people whose experience of their parents was negative have reported increased pain and fear experiences [16]. There may also be incentives to exaggerate psychiatric symptoms, such as securing access to limited services or meeting narrow eligibility criteria for disability benefits in certain countries [17].

Above all, the HiTOP is based on the past literature and questionnaires/scales based on the DSM and the ICD and, in doing so, carry forward some of the major concerns of the past approaches. For example, if questionnaires and scales such as the PTSD checklist for DSM-5 (PCL-5) are designed to collect data consistent with DSM/ICD (to collect data allowing clinicians to ‘score’ patients on various DSM categories, such as PTSD in this case) then using data collected in this way to support the development of an alternative system (e.g. HiTOP) may be problematical because the underlying data are themselves constrained by the DSM. To avoid this problem, in this study, we do not use the scales or questionnaires found across studies that have relied on the DSM or the ICD.

### Addressing the limitations of the emerging taxonomies (researchers’ position)

First, traditional systems consider all mental disorders to be categories. In contrast, the evidence to date suggests that psychopathology exists on a continuum with normal-range functioning [9]. But implementing a pure dimensional approach may be problematic, as it would mean we would need a scale for each symptom, and it might be difficult for pragmatic communication purposes. Therefore, while we focus this study on identifying clusters or categories, we acknowledge that the symptoms might differ in scale/magnitude/frequency. Therefore, we propose that future studies should explore a mid-way. For example, both clusters A and B might have sleep disturbance, but the frequency might differ. This might address some of the DSM’s concerns related to a substantial loss of information and diagnostic instability [18–20]. This is different from the DSM’s approach, where diagnoses can also be mild, moderate, and severe because unlike the DSM, our focus is on individual symptoms (not disorders or category level). Such focus on individual symptoms is likely to be more valid than reliance on human-made constructs such as depression.

Note that the data we used in this study do not have numeric values such as frequency. However, we acknowledge the possibility of symptoms differing in frequency or magnitude here. Therefore, we do not test the assumption and leave that to future studies on this line.

Other than the HiTOP, some taxonomic studies have also proposed non-categorical approaches, such as the Network structure of psychopathology (e.g., [21, 22]) and transdiagnostic approach [23]. In this study, we acknowledge all such possibilities and promises. Still, we attempt to build a categorical model of psychopathology because of its acceptability in the dominant culture and clinical practice and its merits in communication and other value propositions (as mentioned above).

### Importance and aims

This study is an important potential way forward in the classification literature because it discovers potential syndromes in psychopathology based primarily on people’s open-ended narratives about their lived experiences with mental illness. At its least, the current study shows how to create a data-driven bottom-up approach to classifying mental illnesses. The current study aims to group symptoms based on peoples’ lived experiences. The study asks the following questions:

- How many groups or clusters can we group peoples’ experiences with mental illness?
- Which are the top ten symptoms under each of those clusters?
- Which factors or experiences are common to multiple clusters?
- Which factors or experiences are unique to each cluster?
- In how, these clusters are similar?
- How is the density of data distributed between the clusters?
- How well each of these clusters is differentiated from each other?

## Method

We used secondary data, which we gathered and curated for another study [1] which extent are these demonstrate diagnostic heterogeneity between patients diagnosed with Major Depressive Disorder and Bulimia Nervosa. The dataset was used in the current study to address different research questions mentioned above and had personal narratives of 10,933 people who mentioned having received a mental illness diagnosis. In the version of the data used in the current study (i.e., keeping only the symptoms and removing other parts of the sentences), the patients reported an average of 4.8 symptoms with an SD of 3.8. The data can be acquired from https://github.com/Chandril/patient_narratives_processed_data (data-file titled as data29.csv).

### Participants

Ten thousand nine hundred and thirty-three narratives from an online journal (i.e., live journal, https://www.livejournal.com/). LiveJournal is an open social networking service that hosts multiple communities from sports to investing to health (it is not an academic journal as it might seem to some people from the name). The narratives we used were posts made by people who self-reported to have received a psychiatric diagnosis. Anyone can sign-up and share their concerns with the hope that the community will respond with support or advice from other members. There are no moderators. We specifically chose the communities meant for people with a particular psychiatric diagnosis. The members of such communities are currently at different stages of their recovery process. For example, while someone might be in their early stage of illness, others might be those whose condition is either improved or gone (remitting). There are no content or word restrictions. We scraped all the community posts till 16^th^ October 2019 was scraped.

**Fig. 1:**
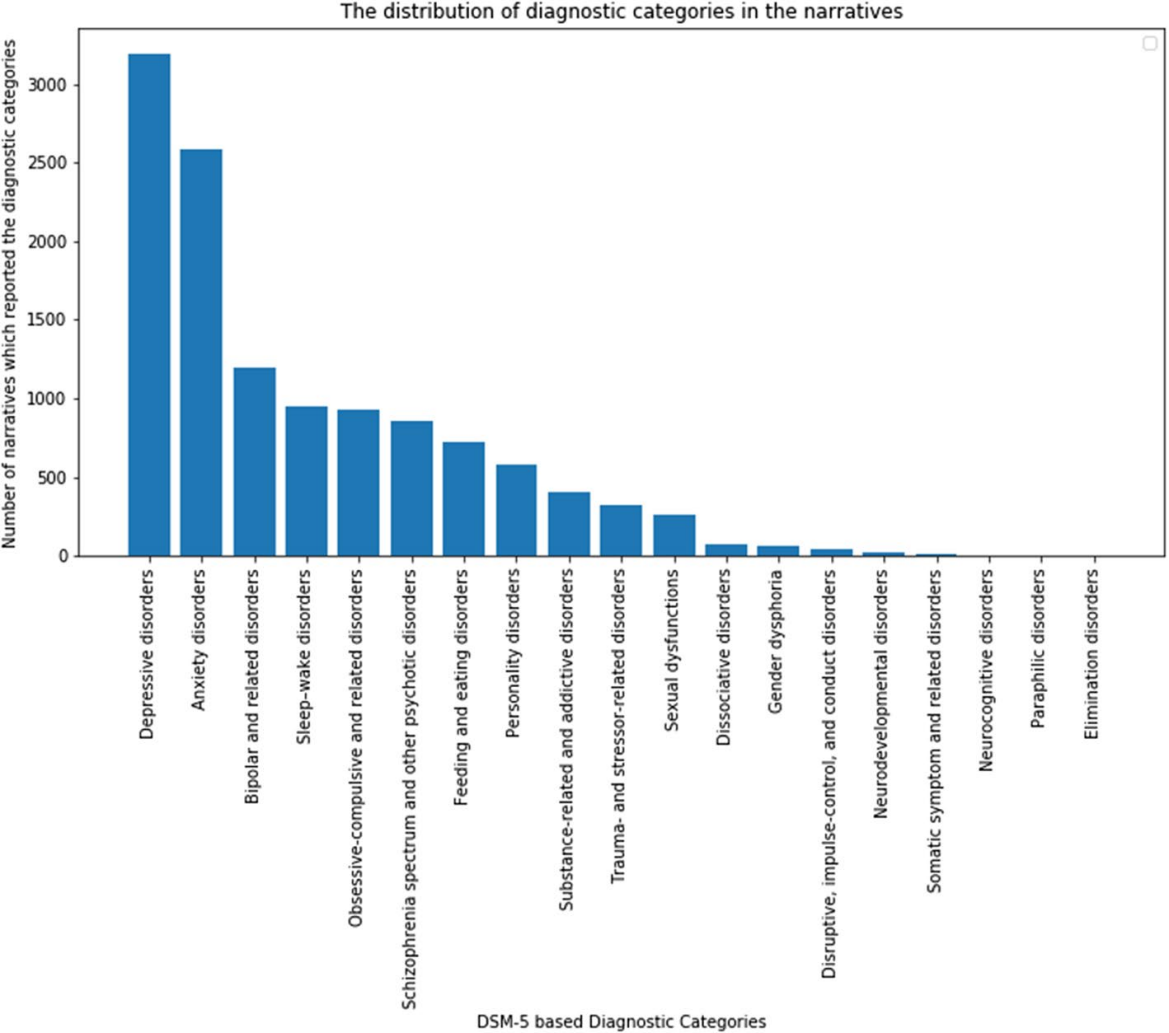
The distribution of diagnostic categories in the narrative sample

The available data do not include the patients’ sociodemographic information nor their geographic location. No directly identifiable data were collected, and narratives were disassociated from usernames before analyses. We collected only English text, indicating that the patient knows how to write in English (inclusion criteria). Also, we know that they were diagnosed with mental illness because they had reported so. It covered 84.2% of all diagnostic categories mentioned in the DSM 5 which is a good range, but it is important to acknowledge that this was not a random, representative sample and so there should be caution about generalising to the wider population and/or across contexts. The distribution of the diagnosis the sample received has been visually presented in Fig. 1. The average number of words in the narratives was 586.5 (Standard Deviation, S.D. = 48.79). As mentioned above, after processing (i.e., keeping only the symptoms/experiences), each patient in our dataset reports an average of 4.8 (~ 5) symptomatic words with an S.D. of 3.82 (~ 4).

From the Fig. 1, it can be suggested that a lot more patients diagnosed with depressive and anxiety disorders wrote narratives on such online platforms than ones with sexual dysfunction and dissociative disorders. Therefore, there is no equal distribution and hence there is a possibility of bias. Our data covered narratives from patients diagnosed with 84.2% of all the diagnostic categories mentioned in the DSM 5. However, the database does not include patients who have explicitly mentioned being diagnosed with neurocognitive disorders (e.g., dementia), paraphilic disorders (e.g. paedophilia), or elimination disorders. Regarding our choice of which words would qualify as “symptoms”—as reported in the study [1]—in which this data was collected, we explained that “while we did refer to the DSM and ICD for gathering collections of words, but we also focused on manual scanning of the words the patients wrote about their mental ill health experiences – without specifying or restricting ourselves to a particular disorder or syndrome (e.g., depression).”

One concern might be that the peoples’ narratives are ‘corrupted’ by DSM given their diagnoses and the wider discourse about the disorder in the media. But that was demonstrated to not be the case in [1] because using the same dataset (as used in the current study), it was found that the patients who received the same diagnosis (e.g., Major Depressive Disorder) reported different symptoms. If their perceptions were shaped by the media or their knowledge of the diagnosis, the study would have found a high similarity between patients who received the same diagnosis.

### The procedure of textual analysis

Pre-processing data: Raw data can be dirty and have missing values, incorrect entries, among several other issues. As reported in a recent study [22], there are 672 unique symptomatic/psychopathological experience-based words in the dataset. Some of them are nonsensible (e.g., “eb”), and some are irrelevant to the context (e.g., “falling”). Therefore, we considered only symptomatic words such as “stress” and “trauma” in our analysis.

Secondly, machine learning algorithm don’t understand the text as well as they understand numbers, so before the data is run through the machine learning algorithm, the text data was converted into numbers (called “vectorisation”). The Term Frequency-Inverse Document Frequency (TF-IDF) vectoriser was used for this purpose. In the current study, the TF-IDF reflects how important a symptom is to the dataset (in a narrative collection). It is often used as a weighting factor. ‘TF’ refers to the information on how often a term appears in a narrative (or document) and ‘IDF’ indicates the information about the relative rarity of a term in the collection of narratives (or documents). Together (TF-IDF), they represent the importance of a “word” being inversely related to its frequency across narratives (or documents). Therefore, in this study, the main purpose of using TF-IDF is to indicate how important a “symptom” is in a patient narrative in a collection of narratives (from all patients combined)—helps to adjust for the fact that some symptoms appear more frequently in a narrative..

This study will use an unsupervised Machine Learning algorithm called K-Means Clustering on the text data to do the clustering (i.e., groups the unlabelled dataset into different clusters). The purpose of using K-Means Clustering is similar to the idea of grouping different elements (e.g., chicken, fruits, and vegetables) and sorting or grouping them based on their similarities and differences (such as vegetarian diet: fruits and vegetables Vs, non-vegetarian diet: chicken). We attempted to group symptoms based on their co-occurring nature in the current study. For example, if low mood and anhedonia tend to co-occur often in the sample and if intrusive thoughts and compulsive actions tend to co-occur often, we expect that the K-Means clustering will form two clusters: one with low mood and anhedonia while the other cluster was having intrusive thoughts and compulsive actions.

In K-Means, K defines the number of pre-defined clusters that need to be created in the process. The K-Means algorithm works iteratively to assign each data point to one of the K groups based on the provided features. For example, in this case, we ran the K-Means Clustering (distance metrics = Euclidean) through 300 iterations (default value) to develop the results. Since k-means have a random initialisation component, multiple instantiations of k-means are often done, and an average is taken, but this is not practical in the current study. So, in most cases, when such averaging is not done, people use the k-means +  + initialisation heuristic. K-Means +  + algorithm helps to improve the conventional initialisation algorithm by choosing the initial values (or “seeds”) for the k-means clustering algorithm. We do not expect the algorithm to carry any form of humanly biasness in this case because it only had data about symptoms and the goal was only to explore or group (not predict X from Y). Had the algorithm been subjected to analyse sociodemographic data, and had the goal been to predict a variable there could have been a possibility of introducing/imitating human bias (e.g., predicting people of black race to be more likely to commit crimes).

After that, the “elbow” method was set to silhouette score (metrics) to select the optimal number of clusters by fitting the model with a range of values for K. The “elbow” method helps us to select the optimal number of clusters by fitting the model with a range of values for K. The default metrics (in the used KElbowVisualizer, [24] is set to distortion (mean sum of squared distances to centres). But we chose silhouette metrics (mean ratio of intra-cluster and nearest-cluster distance) instead. The rationale was that the silhouette coefficient was argued to exhibit a peak characteristic as compared to the gentle bend in the default elbow method [25] which is easier to visualize and reason with.

The elbow method then ran k-means clustering on the dataset for a range of values for k (i.e., 1–10) and then for each value of k computes an average score for all clusters. As mentioned above, these clusters were evaluated using silhouette (mean ratio of intra-cluster and nearest-cluster distance). In the resulting plot, if the line chart resembles an arm, then the “elbow” (the point of inflexion on the curve) is a good indication that the underlying model fits best at that point. Likewise, the best value of silhouette score is 1, and the worst value is -1. Values near 0 indicate overlapping clusters. Negative values generally indicate that a sample has been assigned to the wrong cluster, as a different cluster is more similar.

We chose to report the top ten symptoms under each resultant cluster to make it a practical starting point. We argue that we can put as many symptoms as we want under each cluster but imagine what benefit a taxonomic manual does to a clinician trying to diagnose a patient or a researcher trying to design the treatment—if each of its syndromes has hundreds of symptoms. Finally, we argue that it would be pre-mature to establish these syndromes as biological-truth, as is the case with physical ailments such as cancer or cardiovascular conditions. These are some of the DSM and ICD mistakes. We attempt to explicitly mention those aspects where human decisions were made without pretending to establish the clusters as ground rules. We chose the top 10 symptoms under each cluster for the current study to ensure ease of interpretation and feasibility.

We then used Jaccard’s similarity index as the similarity metric, which measures the similarity between two nominal attributes by taking the intersection of both and dividing by their union. In other words, Jaccard similarity is the number of common attributes divided by the number of attributes that exist in at least one of the two objects. So, the coefficient equals to zero if there are no intersecting symptoms and equals to one if all symptoms intersect. The rationale for choosing Jaccard similarity was associated with a potential problem with the nature of data: narratives often have repetitive words.

As mentioned above, we scrape the user-generated content about their mental health experiences from a social networking service called https://www.livejournal.com/. Time and again, researchers have scraped data from this website and have published it in peer-reviewed journals. An example of one such study that used LiveJournal to scrap the data (concerning mental illness) can be found in [26]. A more generic example of scraping health discussions from websites can be found in [27]. Ethical approval was awarded by the Queen’s Management School Research Ethics Committee. We followed the guidance for internet-mediated research from the British Psychological Society [28] and adhered to copyright laws in conducting this work. Direct consent could not be obtained because of the nature of the data collection. Still, implicit consent was deemed to have been given by virtue of posting in an open forum.

## Result

**Fig. 2:**
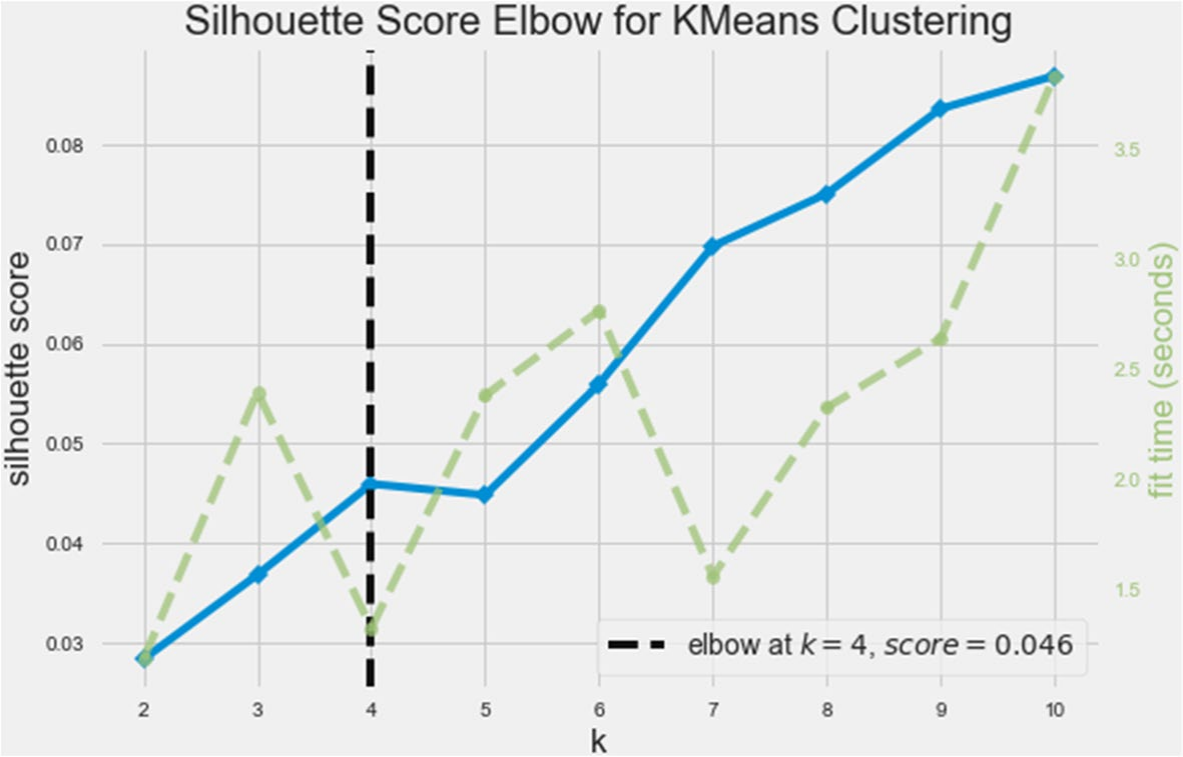
Silhouette score elbow for KMeans Clustering (when the algorithm was run up to a max of k = 10)

The elbow visualiser in Fig. 2 demonstrated that there are four clusters (potential categories of syndromes).

The amount of time to train the clustering model per K is indicated as a dashed green line. This green line is displayed as part of the default KElbowVisualizer’s output (yellowbrick library).

### “There are four clusters of psychopathologies.”

The symptoms and syndromes are distributed in the four clusters when the algorithm was run up to a max of k = 10, as depicted in Table 1. The subsequent analyses will be based on the choice of 4 clusters. It is not arbitrary but based on the point of inflection on the curve suggested by the “knee point detection algorithm” based on the data. This point of inflection is a good indication that the underlying model fits best at that point. The inflection point on the curve (= 4) does not change when the same algorithm is run up to k = 200 (Additional file 1: Appendix A, Fig. 2).

**Table 1:** The distribution of symptoms and syndromes within the four clusters (potential constructs for mental disorders)

Cluster 0:	Cluster 1:	Cluster 2:	Cluster 3:
feeling sick	feeling sick	feeling sick	feeling sick
fear	fear	fear	fear
depressed mood and loss of interest	depressed mood and loss of interest	depressed mood and loss of interest	depressed mood and loss of interest
auditory hallucination	auditory hallucination	auditory hallucination	auditory hallucination
mania and depression			
pain		pain	
experience of loss			
sadness	sadness	sadness	sadness
sleep			sleep
eating			
	repetitive thoughts and actions		
	anxiety	anxiety	anxiety
	compulsion		
	attention deficit		attention deficit
	rituals		
		isolation	
		loneliness	
		cry	
			panic attack
			stress

*Note.* The order or sequence of the symptoms does not matter here. However, the symptoms were arranged in this order to visualise the common and uncommon factors across the clusters

For practical purposes, we have requested the feature extraction module (used in the script, belongs to the sklearn python library) to draw ten words for each cluster. Therefore, each cluster had ten words under them. Note that this decision was taken by researchers based on a pragmatic rationale. This is not to say that there cannot be more than ten signs and symptoms under each syndrome because it is not ground truth. However, we have presented the result for 5 and 20 as well (Additional file 1: Appendix B) for the reader to realise that this does not unduly influence the results and merely adds new symptoms or experiences to the list.

Further, we argue that these syndromes are treated more like human-made constructs built on observed experiences of people who lived through them. Being constructed, these clusters are open to being revised on how many symptoms should be under each syndrome.

We presented ten symptoms or experiences under each cluster to apply and understand the practicality. Note that each cluster label and the numbers associated with the cluster names do not have a meaning. So, cluster 0 can be relabelled as cluster A, cluster 1 as cluster B, and so forth, and they will still mean the same.

#### Transdiagnostic factors

It can be inferred that the following four experiences are common across all the four clusters: feeling sick, fear, depressed mood and loss of interest, and auditory hallucination. Therefore, it can be indicated as transdiagnostic factors. The traditional diagnostic system attempts to group people based on their symptoms. However, we attempted to group the symptoms based on their co-occurrences in the current study. Such generation of clusters of symptoms based on co-occurrences will find its applications in assisting healthcare professionals in guiding their clinical interview questions (e.g., if the patient reports symptom X, then ask about symptom Y, which is co-occurring in the sample).” Therefore, the finding that auditory hallucination presented itself as a transdiagnostic factor does not indicate that the majority of patients experience it. Instead, the finding suggests that auditory hallucination frequently co-occurs with the respective symptoms under each cluster. This possibility was supported/exemplified by the high eigenvalues for these syndromes and symptoms [22]. For example, fear (0.99), auditory hallucination (0.95), depressed mood and loss of interest (0.94) had some of the highest eigenvalues (equal to or greater than 0.80 out of 1.0). A symptom gets a high eigenvalue if it frequently co-occurs with other symptoms. The traditional literature on mental health would probably call such symptoms “comorbid” and “transdiagnostic”. On the other hand, the symptoms with low eigenvalues are the ones that are the distinguishing feature of each cluster.

#### Distinguishing factors among the clusters

Among the unique factors, what distinguishes cluster 0 is the problem with sleep and eating. Cluster 1 can be distinguished by repetitive thoughts and actions, performing rituals (indicative of compulsions). Cluster 2 can be distinguished by the presence of feeling isolated, pain, loneliness and crying spells. Finally, cluster 3 can be distinguished by the presence of panic attacks and stress.

### Heterogeneity within these four clusters

The presence of transdiagnostic factors reflects that there is heterogeneity within our four clusters. Now the next question that arises is to what extent they are overlapping (between clusters). A recent study used Jaccard’s coefficient as a similarity metric on the same narrative dataset [1]. The study aimed to evaluate the heterogeneity within and between the two most homogenous diagnostic categories of Major Depressive Disorder and Bulimia Nervosa. In the current study, we used a similar methodology. Using a simple, open-source online tool (https://planetcalc.com/1664/), we estimated the similarity index between two sets (in this case, each set represented one cluster) with elements in them representing individual symptoms. Entering the two sets (with their respective elements) meant we compare the two clusters and see how many symptoms are identical between them (e.g., cluster 1 Vs cluster 2).

The Jaccard’s coefficients indicate the similarity index between the 4 clusters range from 0.33 to 0.43 (Table 2). The detailed result can be found in Additional file 1: Appendix B (Table 2).

**Table 2:** Test of similarity of narratives obtained within and across clinical diagnoses

	**Cluster 1**	**Cluster 2**	**Cluster 3**
Cluster 0	0.33	0.43	0.42
	(Common conditions: auditory hallucination, depressed mood and loss of interest, fear, feeling sick, sadness)	(Common conditions: auditory hallucination, depressed mood and loss of interest, fear, feeling sick, pain, sadness)	(Common conditions: auditory hallucination, depressed mood and loss of interest, fear, feeling sick, sadness, sleep)
Cluster 1		0.43	0.54
		(Common conditions: anxiety, auditory hallucination, depressed mood and loss of interest, fear, feeling sick, sadness)	(Common conditions: anxiety, attention deficit, auditory hallucination, depressed mood and loss of interest, fear, feeling sick, sadness)
Cluster 2	0.43		0.43
	(Common conditions: anxiety, auditory hallucination, depressed mood and loss of interest, fear, feeling sick, sadness)		(Common conditions: anxiety, auditory hallucination, depressed mood and loss of interest, fear, feeling sick, sadness)
Cluster 3	0.43	0.43	
	(Common conditions: anxiety, auditory hallucination, depressed mood and loss of interest, fear, feeling sick, sadness)	(Common conditions: anxiety, auditory hallucination, depressed mood and loss of interest, fear, feeling sick, sadness)	

*Note.* The table depicts the average Jaccard’s coefficient—either with a diagnosis of Cluster 0, 1, 2 and 3

### Differences in densities within the four clusters

**Fig. 3:**
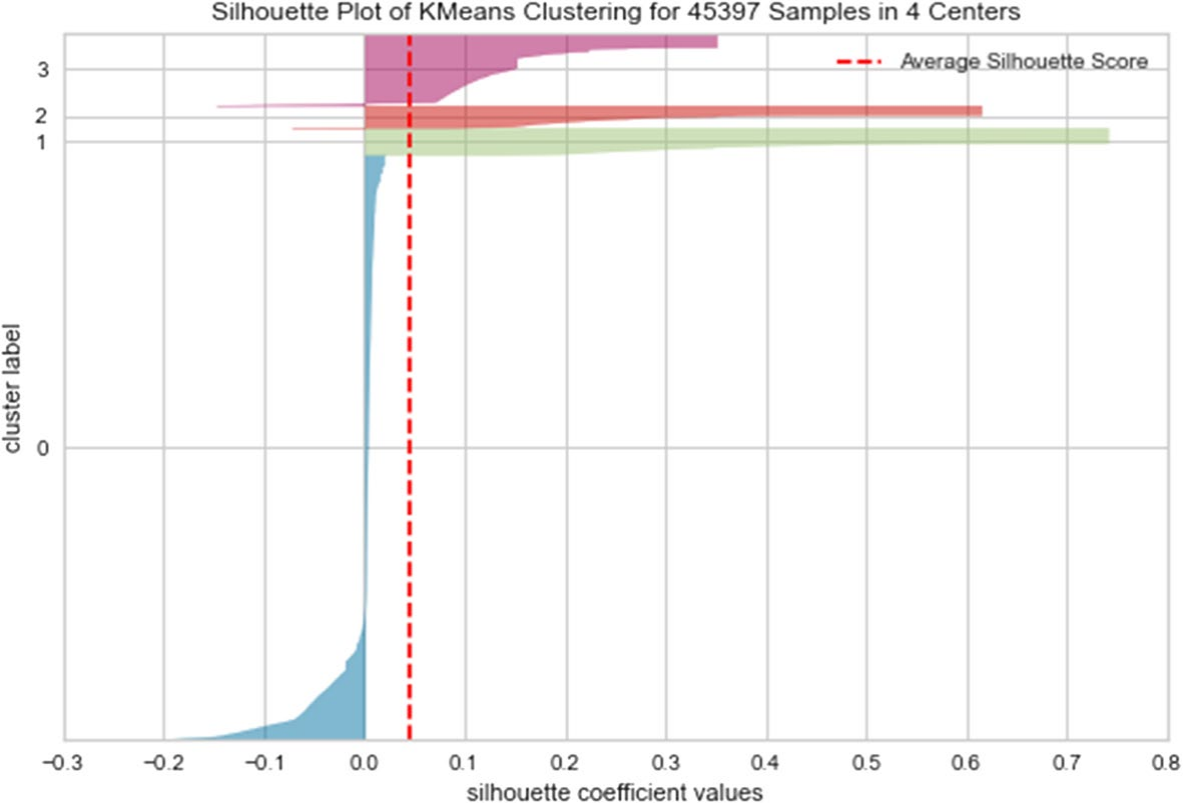
Silhouette plot of KMeans Clustering

The thickness of each silhouette in the plot (Fig. 3) indicates the proportion of the data split into four clusters. The blue colour represents cluster 0, green represents cluster 1, red represents cluster 2, and pink represents cluster 3. Clearly, they are not of equal size. For example, people labelled in cluster 0 have the most heterogeneity and are poorly clustered compared to clusters 1, 2, and 3.

### Uniqueness between the Clusters

In addition to the number of clusters, how they are distributed, and which symptoms are there, it is also important to report how well the clusters are differentiated. The current study used the silhouette value to estimate how similar an object is to its cluster (cohesion) compared to other clusters (separation).

The silhouette ranges from − 1 to + 1. The best value is 1, and the worst value is -1. Values near 0 indicate overlapping clusters. The silhouette coefficients near + 1 indicate that the sample is far away from the neighbouring clusters. A value of 0 indicates that the sample is on or very close to the decision boundary between two neighbouring clusters, and negative values indicate that those samples might have been assigned to the wrong cluster.

As presented in Fig. 1, the current study found the silhouette coefficient to be 0.046, which is closer to zero. This indicates that the four clusters are not well separated or distinguished from one another, indicating reinvention of the diagnostic heterogeneity (comorbidity) between disorders. In turn, hinting that the key problem with DSM might not be the consensus-driven, intuition-based approach it arrives at its classification, but that classification itself is difficult in the context of psychopathology. The elbow method was also tried using the distortion score (Within-Cluster Sum of Scores) as an alternative to the silhouette score (Additional file 1: Appendix A, Fig. 1). However, for both elbow graphs with silhouette and distortion scores, the finding remains the same; that is, there was no distinctive clustering pattern evident in the dataset. So, although we found clusters of symptoms, they are not highly unique in terms of the symptoms. Therefore, it is difficult to differentiate service seekers from each other and make treatment decisions based on these syndromes/clusters.

## Discussion

### The number of clusters

The algorithm generated four syndromes from our dataset. The DSM 5 has 20 disorder chapters, there are specific diagnostic categories, but broadly, the DSM divides people into 20 categories.^1^ Our dataset had patients’ narratives covering 84.2% of the DSM diagnosis (16 out of 19). So, in other words, we can say that when we took narratives from patients diagnosed with 16 different DSM diagnoses, we found 4 clusters. This might indicate that in an attempt to overcome the problem of heterogeneity, the DSM has specified too many disorder subtypes and was stretched way too much. On similar lines, [29] argued that most of such subtypes had been defined rationally rather than derived from structural research and failed to demarcate homogenous subgroups. So, we argue that if there is any attempt to group people into categories of mental disorders based on this dataset, there be four groupings. The extent to which this is generalisable can be verified in future studies using similar but different datasets. In this study, we intended to demonstrate the approach and to draw conclusions about transdiagnostic symptoms.

### Mutually shared factors

The presence of transdiagnostic symptoms such as feeling sick, fear, depressed mood and loss of interest, and auditory hallucination among all the four clusters hints towards the reinvention of the potential problem with the DSM, that is, diagnostic heterogeneity (e.g., [1].

We propose that clusters or syndromes be defined by the symptoms exclusive to the category. Symptoms or experiences that are mutually shared between clusters should be studied to see if they trigger or maintain the unique conditions and the individual differences (e.g., protective factors and social environment) that lead to such differences in mental health trajectories.

### The similarity between clusters

The Jaccard’s coefficients indicate the similarity index between the 4 clusters range from 0.33 to 0.43 (Table 2). This means that about 60–70% of the symptoms are shared between each pair of clusters. Therefore, we found considerable overlap between clusters, which aligns with the idea that a single dimension, called the p-factor, can capture a person’s liability to mental disorder [30]. The possible existence of this general factor of psychopathology (p-factor) has been proposed as it captures the shared variance across psychiatric symptoms. Additionally, it predicts a multitude of poor outcomes and general life impairment. A recent study on this line demonstrates the genetic p factor that represents a continuous, underlying dimension of psychiatric risk [31].

Alternatively, the high overlap can also be taken to be somewhat imitating the DSM’s problem of diagnostic overlap, which might lead a patient to get diagnosed with two different disorders when evaluated by two different physicians. Such low reliability in the current psychiatric diagnostic system is likely to hamper the treatment process of the service seeker. Proponents of the medical model of mental illness argue that comorbidity exists in several recognised medical disorders. For example, individuals with AIDS are relatively likely to develop yeast infections because of their compromised autoimmune system. However, it is undeniable that this leads to problems in diagnosis and treatment. Accepting it on the argument mentioned above would mean *because physical diseases have comorbidity…it is ok if mental illnesses have comorbidities as well*. We argue that future studies and classification approaches must divorce from the categorical viewpoint of mental illnesses and explore alternative viewpoints such as dimensionality and network to understand mental illnesses.

Proponents of the categorical classification model argue that it is required for auxiliary stakeholder decision-making, such as to cover insurance. For example, in the USA, the healthcare system is mostly private and expensive for the service seeker. So, clinicians use DSM-5 diagnoses to request reimbursement from insurance organisations. On that end, we propose that if the primary stakeholders, the patients, do not benefit from this, then keeping the categorical approach for the ease of secondary or tertiary stakeholders (e.g., insurance companies or the government) might not make sense. Instead, research should explore how the insurance can make black-and-white decisions (e.g., Yes/No), such as using disability scales.

### Similarities with the DSM disorders

From Table 3, we can roughly see that some of the clusters are reflecting approximate similarities with the DSM diagnostic manual. For example, eating issues under cluster 0 make it aligned to DSM-5’s Eating Disorders such as Anorexia and Bulimia. Likewise, the presence of repetitive and intrusive thoughts and related actions (e.g., rituals) under cluster 1 is similar to the DSM-5’s Obsessive–Compulsive and Related Disorders. Cluster 2 reflects experiences of depressed mood and loss of interest, sadness, isolation as with patients diagnosed with Depressive Disorders. Finally, the presence of fear, stress, and panic attack indicates an orientation towards anxiety disorders.

**Table 3:** Similarities with existing DSM categories

Clusters mined in the current study from patients’ narratives	Approximate DSM-5 Categories
Cluster 0	Eating Disorders
Cluster 1	Obsessive–Compulsive and Related Disorders
Cluster 2	Depressive Disorders
Cluster 3	Anxiety disorders

### Differences with the DSM disorders

On the other hand, there are marked differences with the DSM in these mined clusters. Most of which are because of the choice of units or conditions to study. The task force of the DSM decided on which symptoms to study and list under each category. In doing so, the task force may have missed thinking about or including symptoms or experiences that were otherwise important.

In contrast, the current study did not have any restrictions. Instead, it mined the free-flowing data written by the patients about their experience with mental illness. In doing so, the current study included numerous words representing experiences that were not present in the DSM’s limited vocabulary. However, the current study acknowledges that the suggestion is not that the symptoms included in the DSM are necessarily inaccurate but more that the way they are currently organised into diagnostic categories does not fully reflect the complexity of people’s experiences. Therefore, the current study also considered the symptoms listed in the DSM.

The inclusion of such words in this study has demonstrated to create a clearer structure of psychopathology. For example, the current study found that people who experience issues with eating might also experience a feeling of loss (cluster A). This raises important questions about the temporal sequence of these two. Does the feeling of loss trigger issues related to eating in some people? Or if it’s the other way round. There is an argument in the popular media that some people use food to self-medicate the pain of loss. Future studies can investigate this possibility.

Other questions such as “are there causal relations between the two symptoms?” (e.g., Is symptom X causing symptom Y)? If so, what makes some people compensate for their feeling of loss with binge eating (for example)? Are there any specific cognitive beliefs or socio-economic and cultural determinants of this trajectory?

Likewise, in cluster 1, we found that people who experience obsessive thoughts and compulsive behaviours might also experience attention deficits. While we do not know which causes the other, but it raises interesting possibilities. For example, will it reduce those symptoms if we apply cognitive training for attention enhancement (e.g., biofeedback) on patients experiencing such intrusive thoughts and compulsions?

### Critical evaluation of one cluster as an example

This attempt to build an alternative taxonomic system of mental illness differs from the previous attempts, such as the DSM and ICD, in one major way. While the DSM and ICD relied on a top-down approach, where the creators of the diagnostic manuals proposed the structure of the disorders and then searched for evidence risking confirmatory bias; in this study, we took an atheoretical approach where we gather lived experiences of about 10,933 people diagnosed with mental illness and clustered their symptoms based on their narratives.

We will review and evaluate cluster A (to provide an example) and draw inferences about the past and future psychopathological nosology. Although we discuss only cluster A here, the same line of thought applies to other clusters too. To prevent repetition and to save space, the current article has discussed cluster A and few others in this discussion section as and when required.

An eating disorder is related to the experience of pain [32]. The cited study found that while 41.2% of the study participants with chronic pain reported that eating disorder symptoms developed after the onset of their pain, 35.3% reported having eating disorder symptoms before they experienced chronic pain. The literature has also associated eating disorders with manic and depressive episodes [33], sleep disturbances [34], self-hatred [35], attention deficit [36], and potential thought-related issues such as cognitive distortions [37] and eating-related intrusive thoughts [38].

On a related note, we offer to treat these clusters (proposed in the current study) as generic themes that warrant further exploration instead of specific conditions. The rationale behind this proposition is that the conceptualisations are based on what patients reported in their narratives and, therefore, based on specific words. So, while we know that there is a problem with weight and sleep in this cluster, we do not know whether the weight increased for some and decreased for others. Likewise, the experience of loss can translate to multiple possibilities, such as loss of control, overeating [39, 40], among other possibilities. Thus, we might require one research project or at least an individual study to investigate each of the 4 clusters.

From the above discussion, we infer two lessons:

- **Reconceptualisation of DSM-based Disorders**
- The traditional nosological systems framed up a construct, such as Eating Disorder. Then other researchers followed it to find associations of Eating disorders with other conditions such as mania, loneliness, and so forth. But we argue that a more data-driven approach to conceptualising psychopathological conditions would be to formulate the construct holistically. Thus, for example, instead of restricting eating disorders to problems related to eating and mentioning all other associated problems as a comorbidity (and hence other disorders), we argue that because the patients experience all these symptoms together (frequently) therefore, the cluster or syndrome for an eating disorder should be reformulated as an accumulation of all these psychopathological experiences (found in this current study and supported by the existing literature).
- **Why does a nosological system need to exist?**
- The points above raise an important question about what is the purpose of a diagnostic system? We argue that it is to group patients’ experiences that frequently co-occur together so that it can guide researchers to design interventions or drugs (e.g., if patients experience X, Y and Z together, then the intervention that is targeted for patients reporting X should also cover for Y and Z) and help clinicians to ask the right clinical questions (e.g., if you experience X, do you also experience Y and Z?).
- We propose that people indicating issues related to their eating should be inquired about their experiences related to pain, sleep and others as indicated by this cluster (cluster A). Accordingly, the purpose of clusters is to assist the clinicians in probing the frequently associated problems that are otherwise important for a treatment plan. Still, the patient might miss, ignore, or forget to report during their primary contact with the clinician.

### Clusters were not distinguishable

Additional evidence to substantiate the unfeasibility of the categorical approach comes from the silhouette score (Fig. 2). One of the criticisms of the DSM was that it was based on the intuitive consensus of a group of people who proposed the categories and not systematic research. Empirical studies, after that, most assumed it to be valid and attempted to further the literature of mental illness. Therefore, the heterogeneity of the DSM can be argued to be the faulty human-led grouping of symptoms. So, in this study, we asked if we attempt to create the categories using patients’ first-hand data (without human intervention), can we find homogenous clusters?

The current study found a silhouette coefficient of 0.046, demonstrating that even when the approach is pure data-driven, the categorical approach does not fit patients’ conditions well into categories. This is not to say that the creation of DSM categories was not arbitrary. That might have been part of the problem, while the other part of the problem is with the categorical ideology of mental illness.

Future studies can collect survey data on those specific experiences (under the syndromes of this study) and then use the quantitative data to build a diagnostic tool using K-Means as a classifier and report the accuracy. If the accuracy falls short, then it can be inferred that even using this ground-up approach to diagnostic classification does not work well. But suppose the accuracy is above 80%. In that case, we can present this as a potential alternative to the existing classification system in psychiatry.

#### Nature of symptoms

The high overlap between clusters clarifies that there are no true clusters or categories of disorders in our sample. But it is interesting to note that we found that symptoms differ by the extent to which they are co-occurring or present across several clusters. Some symptoms, such as Auditory Hallucination, are shared between all 4 clusters. It is consistent with the literature that suggests that hallucinations are prevalent in all DSM-based disorders [41]. On the other hand, symptoms such as trouble with eating are mostly exclusive to one syndrome.

The syndrome of anxiety was found to be present in 3 out of 4 clusters in this study. One reason could be that anxiety is a massive syndrome in itself. Despite people reporting it freely, people might mean different symptoms when they use the term anxiety. So, because of the nature of the data, our findings indicate the words that patients report in this study. However, this calls for an important future study where different anxiety symptoms are to be tested to understand which ones come under which syndrome.

But regardless, in this study, we argue that some symptoms are more exclusive. In contrast, others are generic and present in multiple syndromes. We argue that these trans-diagnostic symptoms (e.g., symptoms within the anxiety syndrome) are prime targets for symptom-level interventions.

### The categorical approach which considers network relations of symptoms with dimensional variations

In the current study, our conceptualisation of psychopathology accepts that symptoms are interrelated as a network, with differing eigenvalues (indicating co-occurrences). But symptoms can also be grouped in different clusters. We argue that the same symptoms can be present in more than one syndrome, but they might be dimensionally different. For example, the frequency of pain experienced might differ in cluster A and someone in cluster E. However, we need additional studies using quantitative data to test this hypothesis. Combined, we attempt to integrate three different approaches (i.e., categorical, network-based, and Dimensional approach) of classification under one system.

### Limitations

The current dataset was based on peoples’ narratives posted or shared online – about their mental health experiences. To make the dataset closer to the real-world psychiatric population, we searched for the narratives of all the possible diagnoses anyone can receive and compiled the narratives of lived experiences to create clusters of symptoms. It is important to acknowledge that the dataset does not represent all types of patients as per the traditional diagnostic system. In other words, we searched for and realised that patients of all diagnoses are not sharing their lived experiences equally. For example, there are conditions such as Narcissistic Personality Disorder (NPD) where the person may not see the problem in themselves. Instead, they complain about the world and others. In such a case, it may be less likely that we will find narratives where people will say, “*I got diagnosed with NPD because I am so self-absorbed and behave negatively to others at times*.”

On the other hand, someone diagnosed with Generalised Anxiety Disorder might write a narrative as “*I was diagnosed with GAD, and I feel scared of everything all the time.*” So, it is unlikely that we will get any narratives from NPD talking about narcissism. Likewise, there maybe more stigma about sexual dysfunction than depression. So, people with sexual dysfunction are less likely to write about it in public forums. However, that being said, there is merit in the current study. The study considered a large sample of 10,933 patients. Therefore may be, it represents the experiences of a certain section of mental healthcare service seekers.

Our sample covers narratives from the patients diagnosed with different disorders, spanning 84.2% of all the diagnostic categories mentioned in the DSM 5. However, our sample lacked any narratives for 3 out of the 19 categories, possibly due to issues of stigma, lack of knowledge, or inability to recall or write memories due to the condition’s nature. Specifically, the database does not include patients who have explicitly mentioned being diagnosed with neurocognitive disorders (e.g. dementia), paraphilic disorders (e.g. paedophilia), or elimination disorders.

Therefore, it is important to note that we are not arguing for a comprehensive taxonomic system based on this current study. Instead, we demonstrate a possible method to create an alternative system. We argue that we collected narratives about the lived experiences of about 10,933 people diagnosed with the traditional psychiatric system, and we found 8 clusters from that data.

A different possibility for the high overlap between clusters could be because of that the fact that the far majority of our sample experienced depressive and/or anxiety symptoms and such overrepresentation of depressive and anxiety symptoms could act as a potential explanation (from a causal perspective) for the high overlap between clusters. That said, the constructs of anxiety and depression are heterogenous in themselves and different questionnaires using different symptoms to assess depression and anxiety indicates that there is little agreement among the experts on them. It might also be that symptoms of anxiety and depression are more consequences of experiencing other psychopathological symptoms (e.g., auditory hallucination triggering anxiety) instead of causing or leading to similar psychopathological experiences between clusters.

The symptoms under each syndrome discovered in this study are based on patients’ narratives. Therefore, by its very nature, there are no numeric data. However, it serves as a qualitative reference point. It requires collecting quantitative data further to investigate the nature of the symptoms’ dimensional distribution (e.g., variations in frequencies under each syndrome).

A further limitation is that many terms are generic because the data was based on what people wrote about their experiences. For example, anxiety forms the most reported and highest eigenvalue. Still, anxiety is not a singular condition. It is a collection of several unique symptoms, as can be seen in the scale items called GAD-7. So, we have a relatively shallow level of understanding using such sources of data. To gain more specific information about which symptoms in anxiety contributed or is related to each syndrome, we need future studies to consider that. However, that being said, the current study is an effort to contribute towards building literature that is divorced from the DSM or ICD based diagnostic categories opening up new possibilities of conceptualising mental illness. The DSM and ICD based categories misled the mental health literature. Many studies use DSM/ICD without acknowledgement or exploration of the limitations of those frameworks. The current study attempts to ward off the tradition and proposes a novel approach to understand mental health diagnostics and highlight the need to revisit why it needs to exist. But at the same time, we acknowledge that our understanding of psychopathology is in its infancy. The current work is far from complete or comprehensive work. Future studies are needed to grow on this line. The academic community needs to understand the nuances of psychopathology somehow.

It is likely that the traditional conceptualisations of mental illnesses, such as the ones proposed by the DSM (and then popularised by mass media), might impact how people perceive, interpret, and tell their mental health stories. However, a recent study [1] using the same dataset demonstrated that the symptoms narrated by the patients who received same diagnosis reported being dissimilar even though DSM likely influences peoples’ narratives (and choice of language).

Studying mental illness will lead to the development of more effective treatment and is expected to help people in crisis. However, harnessing the social network as a data source is a new venture. Therefore, there is still very little guidance available. Few people raise concerns about such data collection sources as being "intrusive", but most people with whom we have sought counsel spoke in favour of using this new source of data, and it was acknowledged that the benefits outweigh any “perceived” concerns – this highlights the need to revisit it. We argue that almost any novel scientific endeavour divides people into two groups: those who support it and those who do not. This is similar to the use of magic mushrooms to treat depression. There are almost always some concerns with the side effects of using psychedelic drugs—still, the scientific communities worldwide progress based on the rationale that the benefits outweigh any potential harm. In our case, we argue that there are no or negligible risks because we accessed only public accounts, no usernames or profiles were recorded, and all words (except the symptomatic experiences) were removed automatically, making it non-traceable to specific individuals – even to the researchers. Also, when someone writes or posts something on an open public forum, the person understands that anyone can read and analyse their content. Therefore, there is implicit consent in the very act of posting an experience online in public. As a result, researchers analysed and drew their inferences from a dataset that had only symptomatic words.

### Future studies

We mentioned the problem of the categorical Vs dimensional approach. The DSM and ICD are categorical, while the evidence suggests that psychopathological experiences are dimensional by nature. We argue that although the evidence goes with the dimensional approach, building a taxonomic system on the dimensional scale of each symptom would indicate each symptom on the scale and no diagnostic system at all to facilitate communication, treatment, prognosis and research. We proposed a mid-way in this study. Signs and symptoms in each syndrome can be dimensional. This means while 2 clusters or syndromes might have “sleep disturbance” as the common symptom. But each of these symptoms might are dimensionally different. For example, for people under cluster A, sleep disturbances happen, maybe seven days a week. But for people under cluster D, sleep disturbance occurs only once or twice a month. Unfortunately, the dataset used in this current study does not have the required quantitative data. Still, it forms a pre-cursor to make such as study possible. Following this study, the immediate next step might be conducting a survey, collecting quantitative data asking about the frequency for specific symptoms under each syndrome we found in the current study and analysing the differences in each symptom’s dimensional aspect under each syndrome.

Such future studies can find the categories via traditional approaches such as Exploratory Factor Analysis (EFA) and Confirmatory Factor Analysis (CFA) studies or via person centred methods like Latent Class Analysis (LCA) and Latent Profile Analysis (LPA) – such lines of investigation would want to know how the categories related to each other and also how they relate to numerous other variables such as behavioural data, how they manifest over time, how they respond to treatment protocols are some more/less resistant and so forth.

Furthermore, future studies can use neural text embeddings as the preferred representation format (instead of the TF-IDF approach) using the same dataset and compare the results from this study.

To conclude, the current study, in a sense, was an attempt to explore the DSM based disorders using clustering patterns from the data given by patients diagnosed with DSM disorders. The test was to see, unguided by the DSM diagnosis but with just the symptoms reported by the patients, how well we can group symptoms into categories. In doing so, we also attempt to demonstrate how can we build alternative forms of diagnostic systems without relying on the traditional ones and thereby avoiding their pitfalls. The hope is to encourage patients’ perspectives to be more central to the mental healthcare’system’s design and delivery.

## Supplementary Information

**Additional file 1:** **Appendix A. Figure 1** Elbow using the distortion score (Within-Cluster Sum of Scores). **Figure 2** Silhouette score elbow for K Means Clustering (when the algorithm was run up to a max of k=200). **Appendix B. Table 1** Potential categories of mental disorders based on patients’ first-hand narratives. **Table 2** Jaccard’s coefficient indicating similarity between clusters. 

## Data Availability

The data can be obtained from Chandril Ghosh (ghoshchandril@gmail.com) upon reasonable request.

## Footnotes

1
*The 20 chapters in the DSM are as follows: Attention-Deficit/Hyperactivity Disorder (ADHD), Autism Spectrum Disorder, Conduct Disorder, Disruptive Mood, Dysregulation Disorder, Eating Disorders, Gender Dysphoria, Intellectual Disability, Internet Gaming Disorder, Major Depressive Disorder and the Bereavement Exclusion, Mild Neurocognitive Disorder, Obsessive-Compulsive and Related Disorders, Paraphilic Disorders, Personality Disorder, Posttraumatic Stress Disorder, Schizophrenia, Sleep-Wake Disorders, Specific Learning Disorder, Social Communication Disorder, Somatic Symptom Disorder, Substance-Related and Addictive Disorders.*
