# Supplementary material for: What can we learn about the psychiatric diagnostic categories by analysing patients' lived experiences with Machine-Learning?
Source: BMC Psychiatry. 2022 Jun 24;22:427. doi: 10.1186/s12888-022-03984-2 (PMC9233399; doi:10.1186/s12888-022-03984-2)
Supplement: Supplementary file 1 — Additional file 1: Appendix A. Figure 1 Elbow using the distortion score (Within-Cluster Sum of Scores). Figure 2 Silhouette score elbow for K Means Clustering (when the algorithm was run up to a max of k=200). Appendix B. Table 1 Potential categories of mental disorders based on patients’ first-hand narratives. Table 2 Jaccard’s coefficient indicating similarity between clusters. [file 12888_2022_3984_MOESM1_ESM.docx]

**Appendix A**

Figure 1. Elbow using the distortion score (Within-Cluster Sum of Scores)


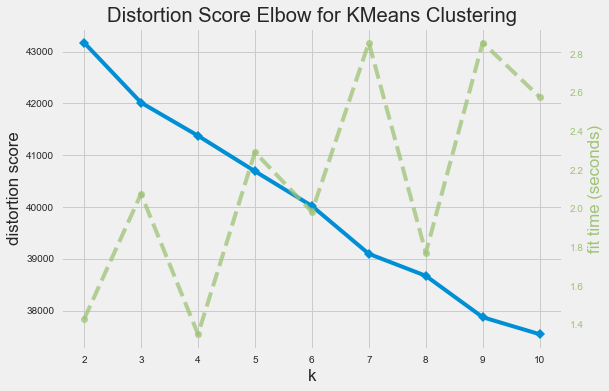


No ‘knee’ or ‘elbow point’ detected. This could be due to bad clustering, no actual clusters being formed, etc.

Figure 2. Silhouette score elbow for KMeans Clustering (when the algorithm was run up to a max of k=200)


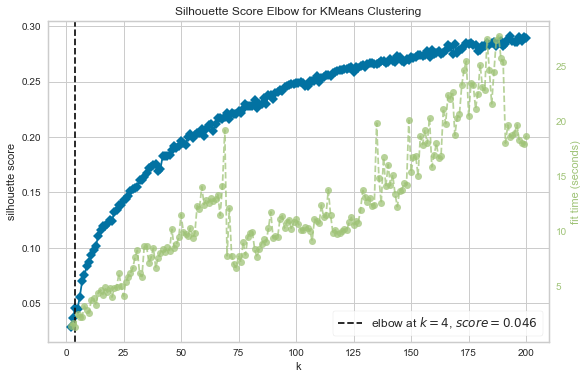


**Appendix B**

Table 1. Potential categories of mental disorders based on patients’ first-hand narratives

| **Clusters with 5 conditions** | **Clusters with 10 conditions** | **Clusters with 20 conditions** |
| --- | --- | --- |
| **Cluster 0:** | **Cluster 0:** | **Cluster 0:** |
| feeling sick | feeling sick | feeling sick |
| fear | fear | fear |
| depressed mood and loss of interest | depressed mood and loss of interest | depressed mood and loss of interest |
| auditory hallucination | auditory hallucination | auditory hallucination |
| mania and depression | mania and depression | mania and depression |
| **Cluster 1:** | pain | pain |
| repetitive thoughts and actions | experience of loss | experience of loss |
| anxiety | sadness | sadness |
| compulsion | sleep | sleep |
| depressed mood and loss of interest | eating | eating |
| fear | **Cluster 1:** | suicide |
| **Cluster 2:** | repetitive thoughts and actions | hatred |
| loneliness | anxiety | attention deficit |
| fear | compulsion | thoughts |
| feeling sick | depressed mood and loss of interest | cry |
| depressed mood and loss of interest | fear | shame |
| anxiety | auditory hallucination | weight |
| Cluster 3: | feeling sick | relationships |
| anxiety | attention deficit | speech |
| sadness | rituals | regret |
| depressed mood and loss of interest | sadness | **Cluster 1:** |
| fear | **Cluster 2:** | repetitive thoughts and actions |
| panic attack | loneliness | anxiety |
|  | fear | compulsion |
|  | feeling sick | depressed mood and loss of interest |
|  | depressed mood and loss of interest | fear |
|  | anxiety | auditory hallucination |
|  | sadness | feeling sick |
|  | isolation | attention deficit |
|  | pain | rituals |
|  | auditory hallucination | sadness |
|  | cry | intrusive |
|  | **Cluster 3:** | thoughts |
|  | anxiety | washing |
|  | sadness | mania and depression |
|  | depressed mood and loss of interest | stress |
|  | fear | eating |
|  | panic attack | disability |
|  | feeling sick | relationships |
|  | stress | speech |
|  | panic | picking |
|  | auditory hallucination | **Cluster 2:** |
|  | sleep | loneliness |
|  |  | fear |
|  |  | feeling sick |
|  |  | depressed mood and loss of interest |
|  |  | anxiety |
|  |  | sadness |
|  |  | isolation |
|  |  | pain |
|  |  | auditory hallucination |
|  |  | cry |
|  |  | shame |
|  |  | speech |
|  |  | suicide |
|  |  | experience of loss |
|  |  | thoughts |
|  |  | relationships |
|  |  | hatred |
|  |  | silence |
|  |  | social |
|  |  | emotional distress |
|  |  | **Cluster 3:** |
|  |  | anxiety |
|  |  | sadness |
|  |  | depressed mood and loss of interest |
|  |  | fear |
|  |  | panic attack |
|  |  | feeling sick |
|  |  | stress |
|  |  | panic |
|  |  | auditory hallucination |
|  |  | sleep |
|  |  | attention deficit |
|  |  | mania and depression |
|  |  | excessive anxiety and worry |
|  |  | thoughts |
|  |  | repetitive thoughts and actions |
|  |  | eating |
|  |  | elevated mood |
|  |  | pain |
|  |  | hatred |
|  |  | shame |

Table 2. Jaccard’s coefficient indicating similarity between clusters

|  | **Cluster 0** | **Cluster 1** | **Cluster 2** | **Cluster 3** |
| --- | --- | --- | --- | --- |
| **Cluster 0** |  | 0.33  (Common conditions: auditory hallucination, depressed mood and loss of interest, fear, feeling sick, sadness) | 0.43  (Common conditions: auditory hallucination, depressed mood and loss of interest, fear, feeling sick, pain, sadness) | 0.42  (Common conditions: auditory hallucination, depressed mood and loss of interest, fear, feeling sick, sadness, sleep) |
| **Cluster 1** | 0.33  (Common conditions: auditory hallucination, depressed mood and loss of interest, fear, feeling sick, sadness) |  | 0.43  (Common conditions: anxiety, auditory hallucination, depressed mood and loss of interest, fear, feeling sick, sadness) | 0.43  (Common conditions: anxiety, auditory hallucination, depressed mood and loss of interest, fear, feeling sick, sadness) |
| **Cluster 2** | 0.43  (Common conditions: auditory hallucination, depressed mood and loss of interest, fear, feeling sick, pain, sadness) | 0.43  (Common conditions: anxiety, auditory hallucination, depressed mood and loss of interest, fear, feeling sick, sadness) |  | 0.43  (Common conditions: anxiety, auditory hallucination, depressed mood and loss of interest, fear, feeling sick, sadness) |
| **Cluster 3** | 0.42  (Common conditions: auditory hallucination, depressed mood and loss of interest, fear, feeling sick, sadness, sleep) | 0.43  (Common conditions: anxiety, auditory hallucination, depressed mood and loss of interest, fear, feeling sick, sadness) | 0.43  (Common conditions: anxiety, auditory hallucination, depressed mood and loss of interest, fear, feeling sick, sadness) |  |

**Note.** The similarity is measured as the ratio of the intersecting set to the union set. If no intersecting elements then Jaccard’s coefficient is 0, but if all elements intersect then Jaccard’s coefficient is 1.
